# Blockade of TGF-β signaling to enhance the antitumor response is accompanied by dysregulation of the functional activity of CD4^+^CD25^+^Foxp3^+^ and CD4^+^CD25^−^Foxp3^+^ T cells

**DOI:** 10.1186/s12967-019-1967-3

**Published:** 2019-07-09

**Authors:** Magdalena J. Polanczyk, Edwin Walker, Daniel Haley, Bella S. Guerrouahen, Emmanuel T. Akporiaye

**Affiliations:** 1 Earle A. Chiles Research Institute, Providence Cancer Center, Portland, OR USA; 2 Veana Therapeutics, Inc., Portland, OR USA; 3 Sidra Medicine, Member of Qatar Foundation, Doha, Qatar

**Keywords:** TGF-β, SM16, Mice, Treg subsets, Anti-tumor response

## Abstract

**Background:**

The pleiotropic cytokine, transforming growth factor (TGF)-β, and CD4^+^CD25^+^Foxp3^+^ regulatory T cells (Tregs) play a critical role in actively suppressing antitumor immune responses. Evidence shows that TGF-β produced by tumor cells promotes tolerance via expansion of Tregs. Our group previously demonstrated that blockade of TGF-β signaling with a small molecule TGF-β receptor I antagonist (SM16) inhibited primary and metastatic tumor growth in a T cell dependent fashion. In the current study, we evaluated the effect of SM16 on Treg generation and function.

**Methods:**

Using BALB/c, FoxP3eGFP and Rag^−/−^ mice, we performed FACS analysis to determine if SM16 blocked de novo TGF-β-induced Treg generation in vitro and in vivo. CD4^+^ T cells from lymph node and spleen were isolated from control mice or mice maintained on SM16 diet, and flow cytometry analysis was used to detect the frequency of CD4^+^CD25^−^FoxP3^+^ and CD4^+^CD25^+^FoxP3^+^ T cells. In vitro suppression assays were used to determine the ability to suppress naive T cell proliferation in vitro of both CD4^+^CD25^+^FoxP3^+^ and CD4^+^CD25^−^FoxP3^+^ T cell sub-populations. We then examined whether SM16 diet exerted an inhibitory effect on primary tumor growth and correlated with changes in FoxP3^+^expression. ELISA analysis was used to measure IFN-γ levels after 72 h co-culture of CD4^+^CD25^+^ T cells from tumor-bearing mice on control or SM16 diet with CD4^+^CD25^−^ T cells from naive donors.

**Results:**

SM16 abrogates TGF-β-induced Treg generation in vitro but does not prevent global homeostatic expansion of CD4^+^ T cell sub-populations in vivo. Instead, SM16 treatment causes expansion of a population of CD4^+^CD25^−^Foxp3^+^ Treg-like cells without significantly altering the overall frequency of Treg in lymphoreplete naive and tumor-bearing mice. Importantly, both the CD4^+^CD25^−^Foxp3^+^ T cells and the CD4^+^CD25^+^Foxp3^+^ Tregs in mice receiving SM16 diet exhibited diminished ability to suppress naive T cell proliferation in vitro compared to Treg from mice on control diet.

**Conclusions:**

These findings suggest that blockade of TGF-β signaling is a potentially useful strategy for blunting Treg function to enhance the anti-tumor response. Our data further suggest that the overall dampening of Treg function may involve the expansion of a quiescent Treg precursor population, which is CD4^+^CD25^−^Foxp3^+^.

## Background

Compelling evidence gathered in recent years indicates that transforming growth factor β (TGF-β) plays a central role in promoting tumor growth, metastasis and invasiveness [1–4]. Due to the broad range of its biological activity, TGF-β acts as both a growth factor for tumor cells [5–7] and a suppressor of immune cell function [8–11]. Emerging evidence indicates that TGF-β produced by tumor cells promotes immunological tolerance [12, 13] via expansion of T regulatory cells (Tregs). The increased frequency of Treg in patients with cancer can be inversely correlated with survival [14–20]. TGF-β is known as one of the key factors responsible for the development and homeostasis of tolerance [2, 21–25]. Thus, it has become apparent that elevated levels of TGF-β in cancer patients and tumor bearing mice may enhance immune tolerance to tumors by expanding the regulatory T cell compartment and directly inhibiting effector cell mechanisms from clearing the established tumor. This leads to tumor evasion and ineffectiveness of immune-based clinical approaches to boost host immunity.

In our previous studies, we demonstrated that blockade of TGF-β signaling using a small molecule TGF-β receptor I antagonist (SM16) inhibited primary and metastatic tumor growth in a T cell dependent fashion [19, 26]. In the current study, we evaluated the effect of SM16 on Treg generation and function. We demonstrate that SM16 abrogates TGF-β-induced Treg generation in vitro *but* does not prevent global homeostatic expansion of CD4^+^ T cell subpopulations in vivo. Instead, SM16 treatment causes expansion of a population of CD4^+^CD25^−^Foxp3^+^ Treg-like cells without significantly altering the overall frequency of Treg in lymphoreplete naive and tumor-bearing mice. Importantly, both the CD4^+^CD25^−^Foxp3^+^ and the CD4^+^CD25^+^Foxp3^+^ T cells in mice receiving SM16 diet exhibited diminished ability to suppress naive T cell proliferation in an in vitro assay compared to Treg from mice on control diet. These findings suggest that blockade of TGF-β signaling is a potentially useful strategy for eliminating Treg function to enhance the anti-tumor response. Our data further suggest that the overall dampening of Treg function may involve the expansion of a quiescent Treg precursor population, which is CD4^+^CD25^−^Foxp3^+^.

## Methods

### Reagents

Fluorescein isothiocyanate (FITC), Allophycocyanin cyanine tandem (APC-H7), R-phycoerythrin (PE) or Allophycocyanin (APC)-conjugated monoclonal antibodies (mAbs) were used for cytofluorometric analysis of anti-mouse Ki67 (BD PharMingen, San Diego, CA, USA), anti-mouse CD4, anti-mouse CD25 and anti-mouse FoxP3 (eBioscience, San Diego, CA). Purified hamster anti-mouse mAbs, anti-CD3 (clone 145-2C11) and anti-CD28 (clone 37.51) were also purchased from BD Pharmingen. Recombinant TGF-β was purchased from Peprotech (NJ, USA). TGF-β neutralizing mAb (1D11) was a gift from Dr. Hong-Ming Hu (Earle A. Chiles Research Institute, Portland, OR). Cell enrichment kits for CD4^+^ and Antigen Presenting Cells (APC, CD90.1^−^) were purchased from MACS Miltenyi Biotec Inc., (Auburn, CA, USA). Dead Fixable Violet Dead Cell Stain Kit was purchased from Invitrogen (L34955, Carlsbad, CA).

### Sm16

SM16 is a novel, orally bioavailable kinase inhibitor that binds to the ATP-binding pocket of TGF-βR1 (ALK5), inhibiting its activation [19, 27, 28]. When tested against a panel of 35 unrelated kinases, SM16 was shown to be highly selective for ALK5 and only moderately inhibited the activity of p38α and Raf [29]. SM16 was kindly provided by Biogen Idec (Cambridge, MA, USA) under a Materials Transfer Agreement. For in vitro studies, SM16 was reconstituted in dimethyl sulfoxide (DMSO) and used at a final concentration of 10 µM. For the oral treatment studies, mice were put on mouse chow containing SM16 (0.45 g SM16/kg food) (Research Diets, New Brunswick, NJ, USA). Control mice were kept on nutrient-matched AIN93G diet.

### Mice

Six to eight weeks old female BALB/c, C57BL/6, Rag^−/−^ knockout mice were purchased from the Jackson Laboratory (Bar Harbor, ME). B6.Cg-FoxP3^tm2Tch/J^ (FoxP3eGFP) were bred in the Animal Facility at the Earle A. Chiles Research Institute (EACRI), Portland, OR. All mice were housed at the EACRI Animal Care Facility in accordance with the Principles of Animal Care (NIH publication no. 85-23, revised 1985). The Institutional Animal Care and Use Committee (IACUC) of the EACRI approved all protocols in compliance with the Guide for the Care and Use of Laboratory Animals. All mice were maintained under specific pathogen-free conditions, fed food and water ad libitum. Mice were routinely checked for any abnormalities until the experiment was terminated. Euthanasia was performed according to guidelines for carbon dioxide asphyxiation when tumor burden was excessive or when mice progressed to a moribund state. No unexpected deaths occurred during this study.

### Tumor cell line and tumor induction

The 4T1 tumor cell line is a poorly immunogenic, highly metastatic variant of 410.4, a tumor subline isolated from a spontaneous mammary tumor that developed in a BALB/cfC3H mouse [30]. The 4T1 tumor was chosen because it is an aggressive TGF-beta-secreting tumor [31] that bears a resemblance to human breast cancer [32, 33]. It was originally kindly provided by Dr. Fred Miller of the Michigan Cancer Foundation (Detroit, MI, USA). The cells were maintained for a limited time in vitro by passage in complete Dulbecco’s modified Eagle’s medium (cDMEM; Lonza, Walkersville, MD, USA), containing 100 U/mL penicillin, 100 mg/mL streptomycin (HyClone Laboratories, Logan, UT, USA), 0.025 mg/mL amphotericin B (HyClone Laboratories) and 10% fetal bovine serum (FBS; Lonza). Trypsin-versene mixture was purchased from (BioWhittaker, MD, USA). For tumor induction, cells were detached using trypsin-versene mixture by incubation for 5 min at 37 °C. Subsequently, cells were harvested, counted, spun and adjusted to a concentration of 1 × 10^6^ cells/mL. For tumor induction, 5 × 10^4^ cells (in 50 µL volume) were injected subcutaneously (s.c.) into the mammary fat pad. When tumors became palpable, mice were randomized to different groups prior to start of treatment.

### Isolation of spleen, lymph node and tumor-infiltrating lymphocytes

Tumor draining inguinal lymph nodes (TDLN) and spleens were resected and pushed through a 70 μm nylon sieve (BD Biosciences Discovery Labware, Two Oaks, CA) to produce a single cell suspension. The cells were then washed (300×*g*, 7 min) and filtered through a 40 μm nylon sieve (BD Biosciences). After red blood cell lysis, the cells were washed (300×*g*, 7 min) and filtered through a 40 μm nylon sieve. To isolate tumor infiltrating immune cells, tumors were resected and minced using a scalpel blade in triple enzyme digest mix containing 10 mg/mL collagenase type IV (Worthington 8 Biochemical Corp. Lakewood, NJ), 1 mg/mL hyaluronidase (Sigma-Aldrich, St Louis, MO), 200 μg/mL DNAse I (Roche Applied Sciences, Indianapolis, IN) in Hank’s balanced salt solution (HBSS, Lonza). The tumors were then incubated with agitation (37 °C, 45 min). After addition of ethylenediaminetetraacetic acid (EDTA, 10 mM), the digest was incubated for another 15 min. Subsequently, the digested tissue was pushed sequentially through 70 μm and 40 μm nylon sieves, washed (300×*g*, 7 min), overlaid on Ficoll (FicoLite-LM, Atlanta Biologicals, Lawrenceville, GA) and centrifuged (1500×*g*, 25 min, without brake). The interface was collected and washed twice (300×*g*, 7 min).

### Cell enrichment

For purification of CD4^+^ T cells, single cell suspensions of splenocytes were enriched untouched by immunomagnetic bead selection using MACS Miltenyi system (Miltenyi, Auburn, CA, USA) according to the manufacturer’s protocol Briefly, CD4^+^ T cells were enriched by depletion of magnetically labeled contaminating CD8α^+^, CD11b^+^, CD11c^+^, CD19^+^, CD45R (B220)^+^, CD49b (DX5)^+^, CD105^+^, MHC-class II^+^, and Ter-119^+^ (erythroid) cells. The highly enriched (90%) CD4^+^ T cells were subsequently stained with anti-CD4 and anti-CD25 mAbs, and, depending on the cell donor, either CD4^+^CD25^+^ cells (BALB/c), or CD4^+^CD25^+^FoxP3^+^ (FoxP3eGFP) cells were sorted from CD4^+^CD25^–^ or CD4^+^CD25^–^FoxP3^−^ cells respectively, using a FACSAria sorter (BD Immunocytometry Systems, San Jose, CA). In some experiments, CD4^+^CD25^–^FoxP3^+^ cells were also enriched. Naive mice served as donors of responder T cells (CD4^+^CD25^–^ or CD4^+^CD25^–^GFP^–^) and CD4^+^ enrichment was done as described above. In addition, splenocytes from naive female mice were also used to prepare APC. Briefly, cells were incubated on ice for 15 min with anti-CD90.1 PE-conjugated microbeads. After wash, cells were sorted using the MACS magnetic column. The negative fraction (CD90.1^−^) was washed, re-suspended at 1 × 10^6^/mL in cRPMI and irradiated with 30 Gy in a cesium irradiator. The overall purity of T cell-depleted APC was ~ 80%.

### Generation of induced Treg in vitro

For the in vitro induction of Treg cells, 6-well plates were coated with anti-CD3 and anti-CD28 mAbs 24 h prior to the assay in PBS at 4 °C. Two different concentration of Abs were used to induce the activation of naive T cells and they were respectively: 2 µg of CD3/CD28 per mL or 10 µg of CD3 and 3 µg of CD28 per mL. Subsequently, plates were rinsed 3 times with fresh PBS and 1 × 10^6^ FACSAria enriched CD4^+^CD25^–^ or CD4^+^CD25^–^GFP^–^ T cells were seeded in the plate in the presence of 2 ng/mL of recombinant TGF-β for 72 h in cRPMI. The TGF-β neutralizing Ab (1D11) or SM16 was added to some cultures. After 72 h, cells were harvested and washed once in cRPMI. The cell pellet was reconstituted in 0.2 mL of cold PBS and FoxP3 expression was determined after antibody staining.

### Adoptive T cell transfer

For adoptive transfer, MACS-enriched CD4^+^ spleen cells from FoxP3eGFP donors were sorted into CD4^+^CD25^–^GFP^–^ and CD4^+^CD25^+^GFP^+^ populations. An average of 5 × 10^6^ CD4^+^CD25^–^GFP^–^cells were transferred i.v. into each Rag^−/−^ recipient. Upon injection, mice were separated into two groups and maintained either on vehicle or SM16 diet for up to 21 days.

### Flow cytometry

Five-color (FITC, PE, APC, APC-H7, PB) flow cytometry analysis of stained cells was performed to determine FoxP3^+^ cell frequency, as well as the phenotype and proliferation of cells isolated from lymph node, spleen and tumor in untreated and SM16-treated mice. Briefly, pooled cells were divided equally (1 × 10^6^ cells per tube) and washed with PBS and pre-incubated for 30 min at 4 °C with 1 µL of live/dead cell stain kit. Subsequently, cells were washed twice in staining buffer, and cells isolated from tumors were pre-incubated additionally with anti-mouse CD16/CD32 (FcBlock, BDPharMingen) mAb to block non-specific binding to Fc receptors and extracellular staining for CD4, CD25 was performed for 25 min on ice. After incubation, cells were washed three times with staining medium and intracellular staining for FoxP3 and Ki67 was performed using a modified eBioscience protocol. Briefly, cells were fixed for 45 min and washed twice with 2 mL of eBioscience permeabilization buffer. The cells were stained for 15 min with Fc-Block followed 10 min later with fluorescent-labeled antibodies to Ki67, FoxP3 or proper isotype control. The cells were then washed twice with 2 mL of permeabilization buffer, re-suspended in staining buffer and run on an LSRII Flow cytometer. Data were analyzed on FlowJo software (TreeStar, Inc., Ashland, OR, USA). Data represent 10,000 gated CD4^+^ T cells unless otherwise noted.

### Treg suppression assay

After FACSAria sort of MACS-purified CD4^+^ T cells, the enriched negative fraction of either CD4^+^CD25^–^ or CD4^+^CD25^–^GFP^–^ was washed, re-suspended at 1 × 10^6^/mL in cRPMI. The CD90-depleted irradiated splenocytes of naive mice served as source of APCs. For in vitro assays, cells were re-suspended in complete Roswell Park Memorial Institute 1640 medium (cRPMI 1640) containing 100 U/mL penicillin, 100 mg/mL streptomycin (HyClone Laboratories), 0.025 mg/mL amphotericin B (HyClone Laboratories), 70 μM β-2-mercaptoethanol (Sigma-Aldrich), 2 mM l-glutamine (Lonza), 1 mM sodium pyruvate (Lonza), 1 × nonessential amino acids (Lonza), 10 mM HEPES (4-(2-hydroxyethyl)-1-piperazineethanesulfonic acid; Lonza) and 10% FBS (Lonza). Suppression assays were performed in 96-well round-bottomed plates (Becton–Dickinson) in a final volume of 200 µL per well of cRPMI. Both APC and responder cells (CD4^+^CD25^–^ or CD4^+^CD25^–^ FoxP3^–^) were plated at 0.5 × 10^4^ cells per well in triplicates and suppressor cells (CD4^+^CD25^+^, CD4^+^CD25^+^FoxP3^+^ or CD4^+^CD25^–^FoxP3^+^) were added at the following responder to Treg ratios: 1:0, 1:1, 1:0.5, 1:0.25, 1:0.1 and 0:1. Anti-CD3 antibody was added to each co-culture at a final concentration of 1 µg per mL. Co-cultures were incubated for 48 h and [^3^H]-thymidine was added during the last 12–18 h of culture. Subsequently, the cells were harvested on glass fiber filters and assessed for uptake of the labeled thymidine by liquid scintillation counting.

### IFN-γ ELISA

For in vitro assay of IFN-γ secretion, co-cultures of suppressors and responders were plated at 1:1 ratio in a 48-well plate. Wells containing only suppressor or responder cells were included as controls. Cell-free supernatants were harvested after 72 h and stored frozen at − 20 °C until the cytokine assay was performed. The amount of IFN-γ was evaluated in triplicate using the OptEIA ELISA kit (Pharmingen). A standard curve was generated to evaluate concentrations of IFN-γ in the samples using GraphPad Prism version 5b software (GraphPad Software, La Jolla, CA, USA).

### Statistical analysis

Statistical significance of differences among data sets of treatment groups was assessed by Student’s t-test for pair-wise comparisons or for comparisons of multiple groups by one-way analysis of variances (ANOVA) with Tukey’s HSD test to adjust for multiple comparisons. All analyses were performed using Prism software (GraphPad, San Diego, CA). Probability values (P) of < 0.05 were considered indicative of significant differences between data sets.

## Results

### SM16 inhibits de novo Treg generation in vitro

**Fig. 1:**
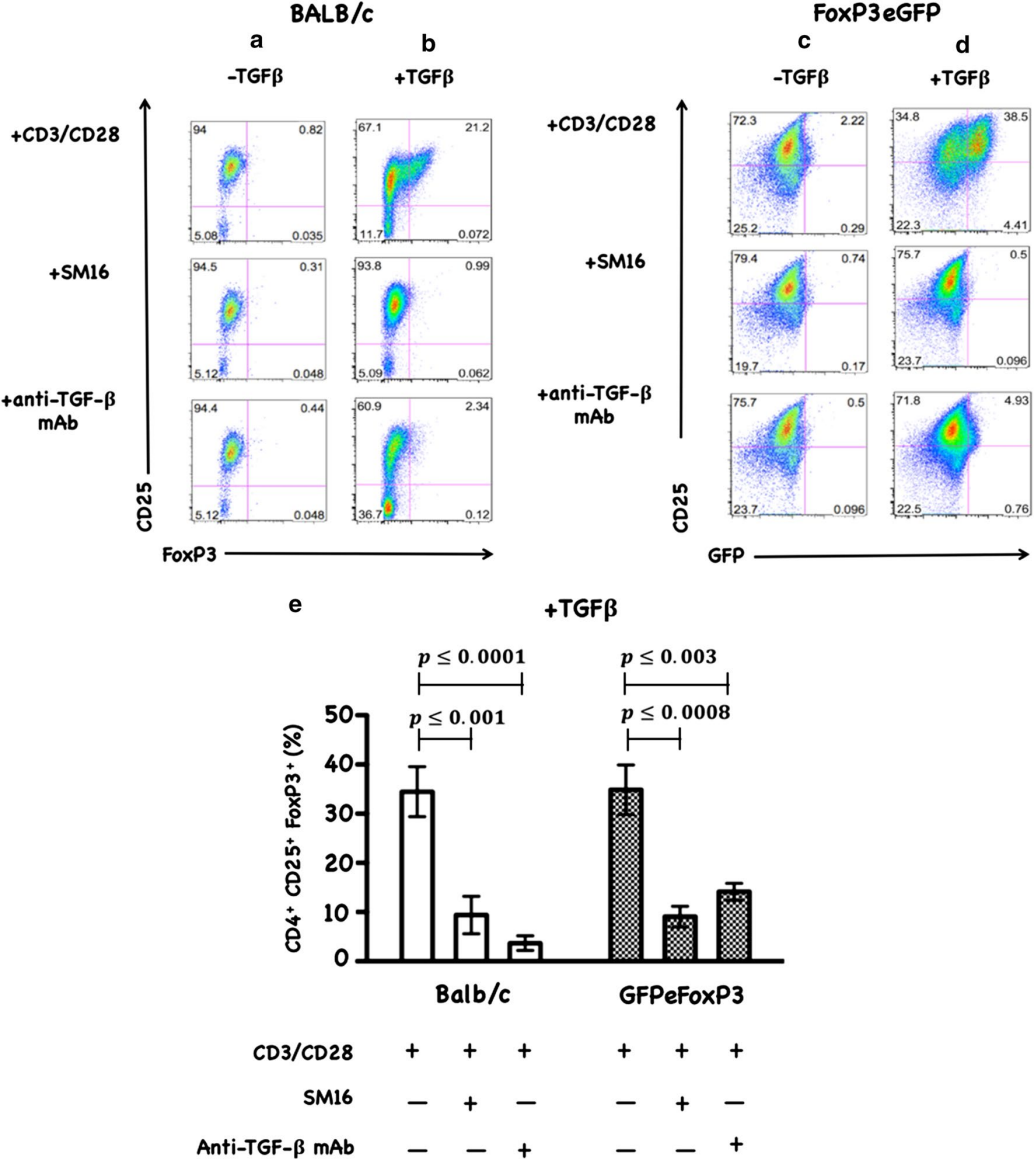
FACS data of CD4^+^CD25^−^ or CD4^+^CD25^–^FoxP3^−^ T cells from spleen of BALB/c and FoxP3eGFP mice. Cells were treated in vitro for 72 h with CD3/CD28 antibodies as described in the “Methods” section. Plots show CD25-PE on the y-axis and FoxP3-APC or FoxP3eGFP on the x-axis. Quadrant statistics noted are percent of live CD4^+^ gate. **a**–**d** Mean values of % CD4^+^CD25^+^Foxp3^+^ from 5 independent experiments show SM16 effects on TGF-β-induced Treg conversion (**e**). Significance between control and experimental groups were determined by Student’s t-test (*, P < 0.05)

To determine whether the mechanism by which SM16 induces anti-tumor response involves the inhibition of de novo Treg generation, we first tested its capacity to block TGF-β-induced Treg generation from in vitro CD3/CD28-activated CD4^+^CD25^–^ and CD4^+^CD25^–^GFP^–^ T cells from BALB/c and FoxP3eGFP donors, respectively. As demonstrated in a representative example (Fig. 1), TGF-β induces Foxp3 expression in CD3/CD28 activated T cells (1A, 1D). Treatment with SM16 or TGF-β neutralizing antibody (anti-TGF-β mAb) abrogated TGF-β -induced conversion to FoxP3 positive cells (1B, 1D). TCR activation in the absence of TGF-β did not stimulate FoxP3 expression (1A, 1C). Repeat independent experiments confirmed SM16 blockade of TGF-β-induced Treg conversion (1E). Similarly, in vitro stimulation of naive CD4^+^CD25^–^ cells from transgenic mice expressing a dominant negative form of TGF-β receptor type II [34] did not result in Treg conversion (*data not shown*).

### SM16 enhances homeostatic proliferation and induces a population of CD4^+^CD25^–^FoxP3^+^ cells in vivo

**Fig. 2:**
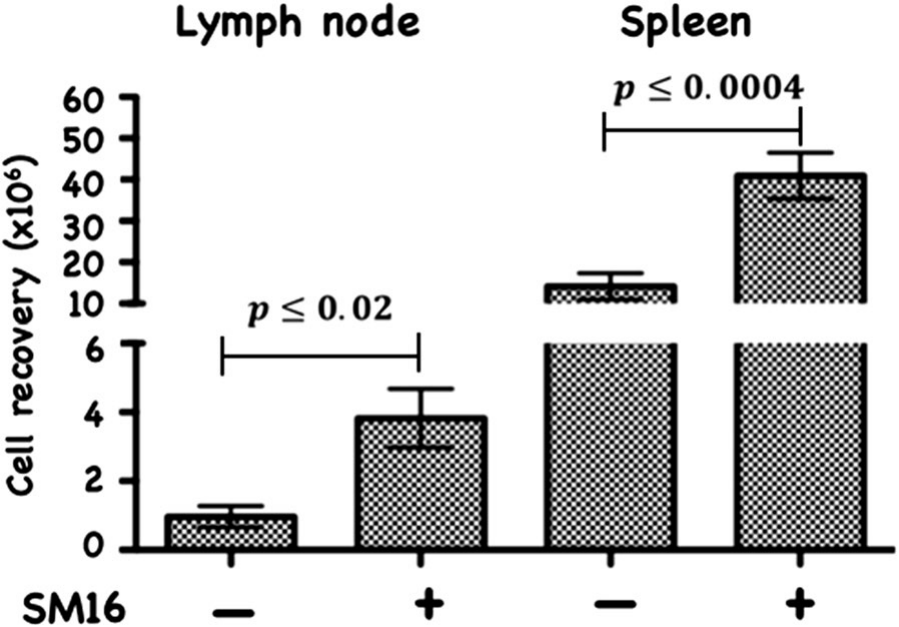
Total CD4^+^ T cell recovery from lymph node and spleen of RagKO mice reconstituted with CD4^+^GFP^−^ cells from FoxP3eGFP donors. The cells were isolated from recipients 21 days after cell transfer and treatment with SM16. Control mice were maintained on standard diet. Data presented are mean of 4 independent experiments. Significance between control and experimental groups were determined by Student’s t-test

**Fig. 3:**
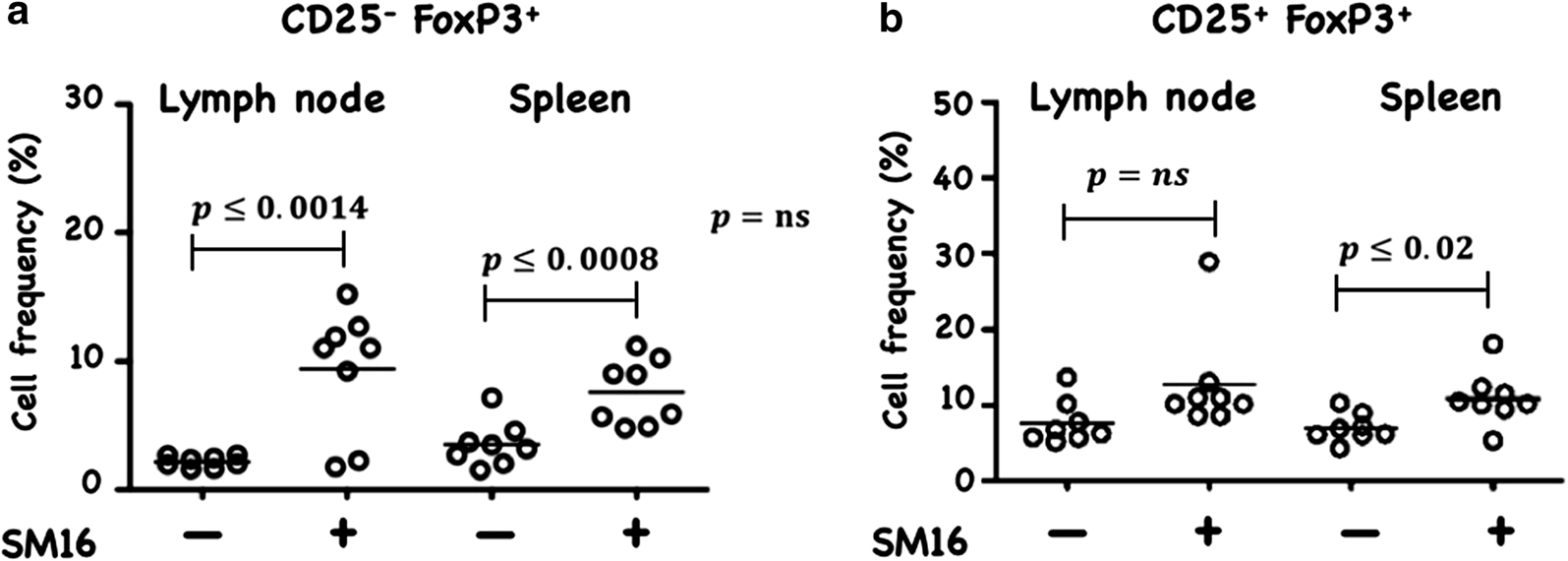
Frequency of CD25^−^FoxP3^+^ and CD25^+^FoxP3^+^ (Treg) cells, respectively among CD4^+^ cells isolated from lymph node and spleen. Naive FoxP3eGFP mice were maintained for 14 days on control or SM16 diet. Subsequently, mice were sacrificed, and lymph node and spleen were isolated to perform flow cytometry analysis. Frequency of CD25^−^FoxP3^+^ (**a**) and CD25^+^FoxP3^+^ Tregs (**b**) cells. Data presented are mean values from eight animals in 2 independent experiments. Significance between control and experimental groups were determined by Student’s t-test

The blockade of TGF-β-induced Treg generation by SM16, was not unexpected as many studies have documented the key role of TGF-β signaling on induction and maintenance of Treg [2, 21–25]. However, this is the first report to demonstrate inhibition of FoxP3 expression among CD4^+^CD25^−^FoxP3^−^ cells in vitro by a small molecule TGF-βRI signaling inhibitor. We next wanted to determine if SM16 could also prevent Treg generation in vivo. We investigated the effectiveness of systemic blockade of TGF-βRI signaling on de novo Treg generation in vivo in adoptive transfer studies using RagKO mice reconstituted with CD4^+^GFP^–^ T cells from FoxP3eGFP donors. As shown in Fig. 2, all reconstituted mice maintained on SM16 diet demonstrated a statistically significant increase in total CD4^+^ cellularity in the lymph node and spleen. Calculation of the absolute cell numbers of different T cell populations revealed a statistically significant increase in Treg cells (CD4^+^CD25^+^FoxP3^+^), as well as activated (CD4^+^CD25^+^FoxP3) and naive (CD4^+^CD25^–^FoxP3^–^) T cells in both LN and spleen (Table 1). Since the adoptive transfer studies suggested that SM16 might induce global T cell proliferation, we further tested its effect on endogenous T cell proliferation in lymphoreplete mice. SM16 treatment resulted in increased frequency of CD4^+^CD25^−^FoxP3^+^ T cells from gated CD4^+^ T cells in both spleen and lymph node (Fig. 3a). CD4^+^CD25^+^FoxP3^+^ T cells were also significantly increased in spleen cells, and trended higher in lymph node cells (Fig. 3b).

**Table 1:** The absolute cell number of CD4^+^ T cell subsets calculated based on flow cytometry analysis of lymph nodes and spleen of RagKO mice reconstituted with CD4^+^GFP^−^T cells and maintained on control or SM16 diet for 21 days

	Absolute cell number per mouse (10^5^)			
	Lymph node		Spleen	
Treatment	Control (%)	SM16 (%)	Control (%)	SM16 (%)
Total cell recovery				
All CD4^+^	1.18 ± 0.5	13.5 ± 1.4	21.7 ± 0.05	88.8 ± 1.90
CD4^+^ CD25^+^ Foxp3^+^ (Treg)	0.1 ± 0.06 (1.4)	0.4 ± 0.03* (3.6)	0.4 ± 0.2 (3.7)	2.5 ± 1.03^a^ (3.8)
CD4^+^ CD25^+^ Foxp3^−^ (activated T cell)	0.08 ± 0.04 (8.3)	0.7 ± 0.04^#^ (6.4)	1.6 ± 1.3 (3.9)	2.5 ± 0.7^b^ (2.7)
CD4^+^ CD25^−^ Foxp3^−^ (other)	1.0 ± 0.5 (91.3)	12.4 ± 6.3^&^ (90.0)	19.7 ± 0.01 (94.0)	83.8 ± 0.01^c^ (93.5)

Absolute numbers (AN) are presented as the mean trait value ± SEM. The % was determined based on average of ANs from 4 independent experiments

*p value ≤ 0.05, as compared to RagkO on control diet

^#^*p* value ≤ 0.05, as compared to RagkO on control diet

^&^*p* value ≤ 0.05, as compared to RagkO on control diet

^a^*p* value ≤ 0.05, as compared to RagkO on control diet

^b^*p* value ≤ 0.05, as compared to RagkO on control diet

^c^*p* value ≤ 0.05, as compared to RagkO on control diet

### SM16 reduces suppressive activity of CD4^+^CD25^+^FoxP3^+^ and CD4 + CD25^−^FoxP3^+^ T cells

**Fig. 4:**
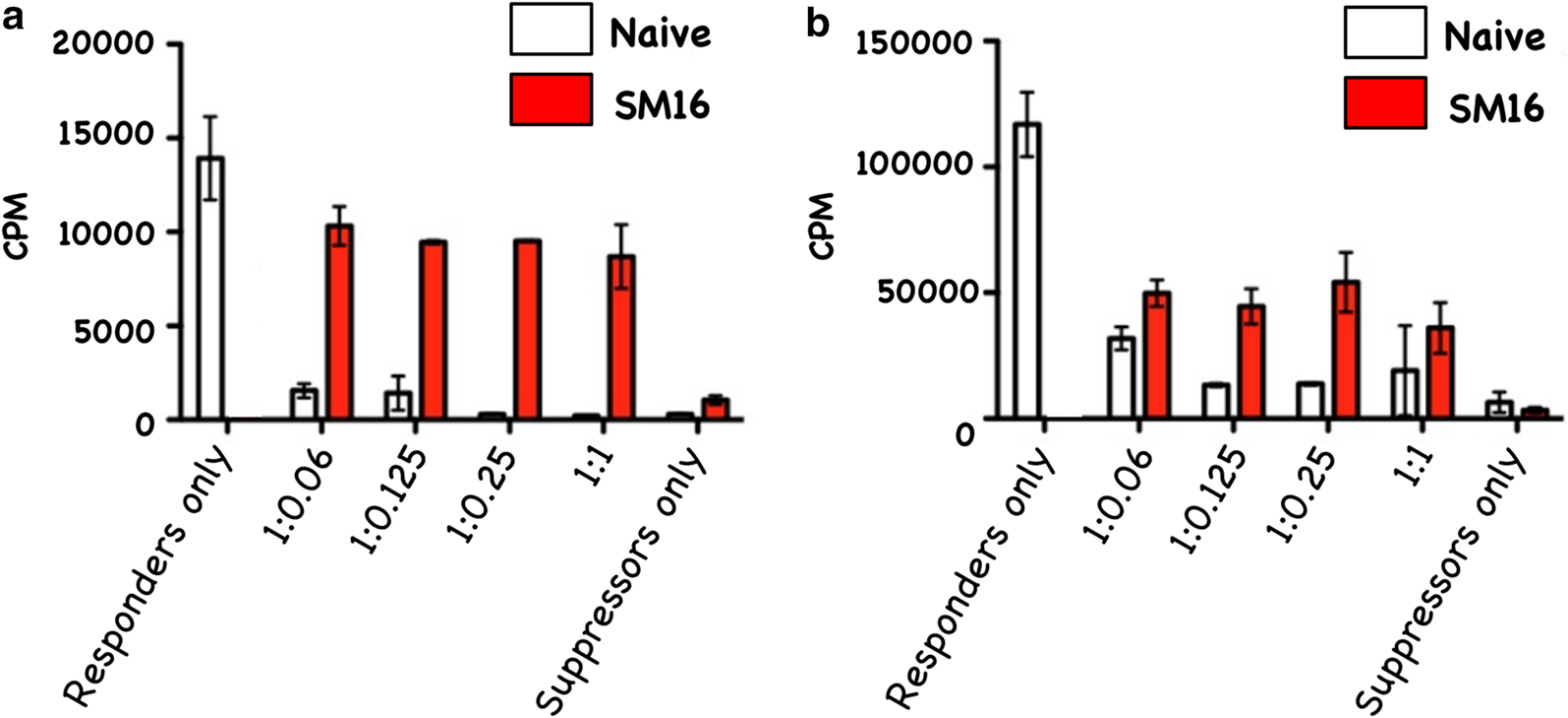
In vitro suppressive activity of CD4^+^CD25^+^FoxP3^+^ (**a**) and CD4^+^CD25^−^FoxP3^+^ (**b**) T cells isolated from naive or SM16 treated FoxP3eGFP mice. CD4^+^CD25^+^FoxP3^+^ and CD4^+^CD25^−^FoxP3^+^ cells from donor mice were obtained by FACS sorting. The activity of isolated cells having the ability to suppress naive T cell proliferation in an in vitro assay was tested by adding increasing numbers of CD4^+^CD25^+^FoxP3^+^ or CD4^+^CD25^−^FoxP3^+^ T cells to co-cultures of sorted CD4^+^CD25^−^ (responder cells) and APCs from naive mice in the presence of anti-CD3. After 48 h the plates were pulsed for 18 h with [^3^H]-thymidine. Subsequently, the cells were harvested on glass fiber filters and assessed for uptake of the labeled thymidine by liquid scintillation (4 and 3 independent experiments respectively). Repeat assays of CD4^+^CD25^+^Foxp3^+^ cells were performed using a single batch of naive responder mice. Similarly, repeat assays of CD4^+^ CD25^−^Foxp3^+^ cells were performed using a separate batch of responder cells from naive mice

Although our in vivo data demonstrated that SM16 did not prevent de novo Treg generation, but instead induced global expansion of CD4^+^ T cells, we further sought to determine whether SM16 treatment had an impact on the function (suppressive activity) of Treg. As shown in Fig. 4a, CD4^+^CD25^+^FoxP3^+^ T cells enriched from spleen of mice on control diet were significantly more efficient at suppressing thymidine uptake by CD3-stimulated responder cells compared to mice on SM16 diet. Similarly, CD4^+^CD25^−^FoxP3^+^ T cells enriched from SM16-treated donors demonstrated reduced suppressive activity as compared to their counterparts on control diet (Fig. 4b).

### SM16 expands FoxP3^+^ cells in tumor bearing mice

**Fig. 5:**
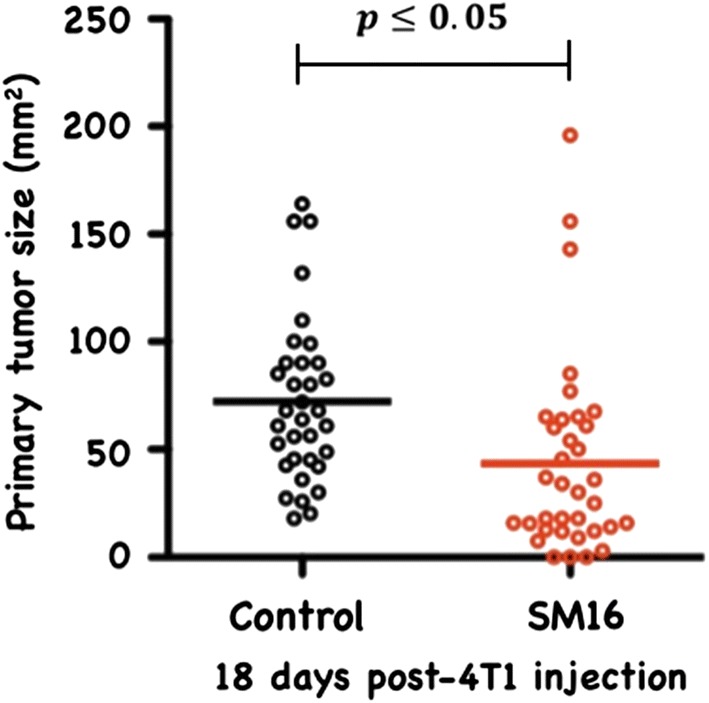
Primary tumor size in SM16-fed and control mice. Data represent average tumor size from all animals in 3 independent experiments. Significance between control and experimental groups was determined by Student’s t-test

**Fig. 6:**
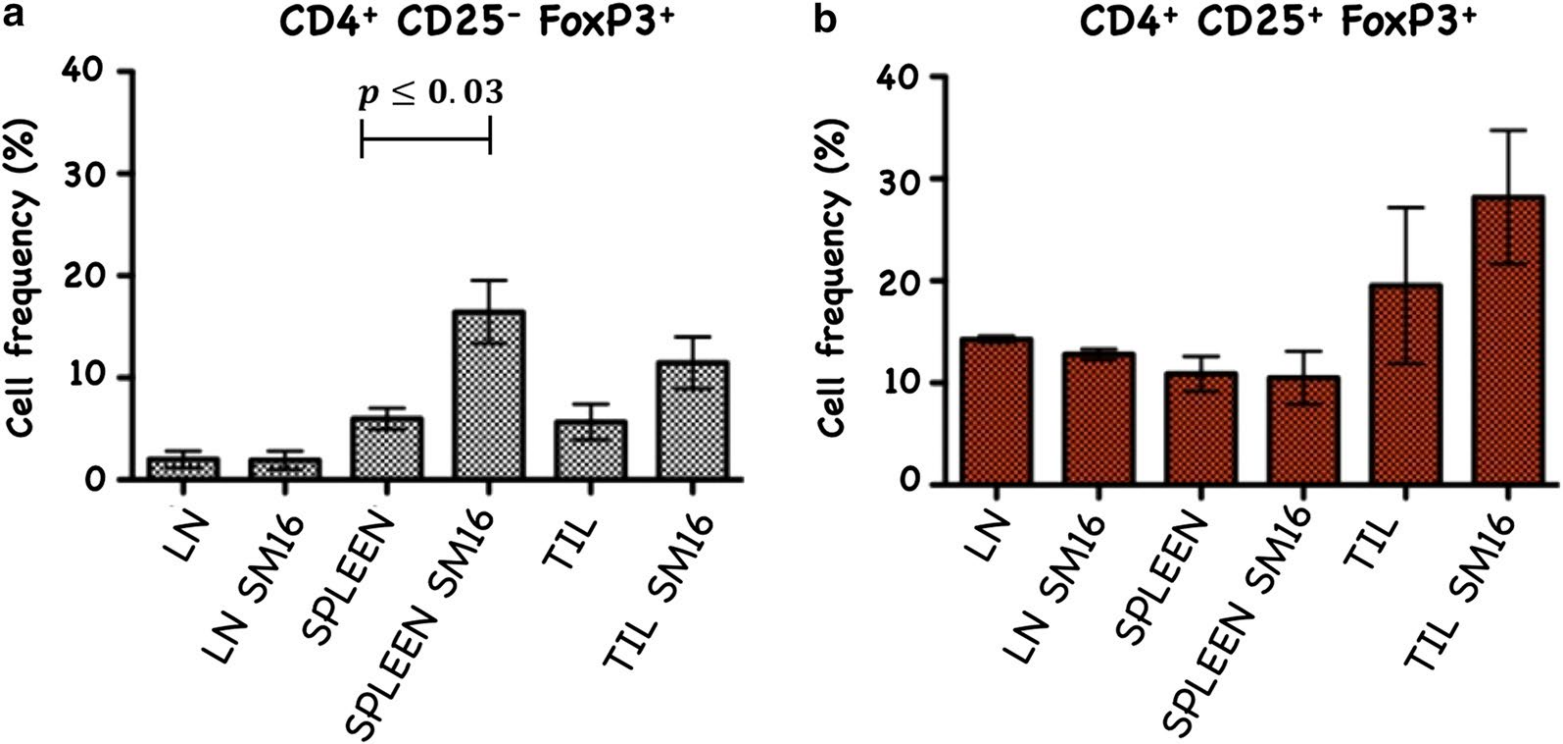
Frequency of CD4^+^CD25^−^FoxP3^+^ (**a**) and CD4^+^CD25^+^FoxP3^+^ T cells (**b**) in lymph node, spleen and TIL. Cells were stained with anti-mouse CD4 and anti-mouse CD25 and after permeabilization, cells were stained additionally with FoxP3. Data represent average of 3 independent experiments. Significance between control and experimental groups were determined by Student’s t-test

We previously demonstrated that 0.45 g/kg-dose of SM16 dramatically reduces the growth of primary and metastatic 4T1 tumors [19]. To determine whether the beneficial anti-tumor effects of SM16 were associated with changes in the Treg compartment, we analyzed the frequency of *FoxP3*^+^ *cells* in the presence or absence of SM16 in a tumor prevention model in which mice receiving injection of 4TI cells into the mammary pad were immediately put on control or SM16 diet. As in our earlier study, we again showed this dose of SM16 significantly inhibited tumor growth (Fig. 5). The inhibitory effect of SM16 on primary tumor growth was associated with a significant increase in CD4^+^CD25^–^FoxP3^+^ in the spleen but not in the tumor draining lymph nodes or tumor (Fig. 6a). Notably, there was no increase in CD4^+^CD25^+^FoxP3^+^ T cells in all studied tissues in tumor-bearing (TBM) mice on SM16 diet (Fig. 6b).

### SM16 attenuates the suppressive activity of Treg in mice bearing established tumor

**Fig. 7:**
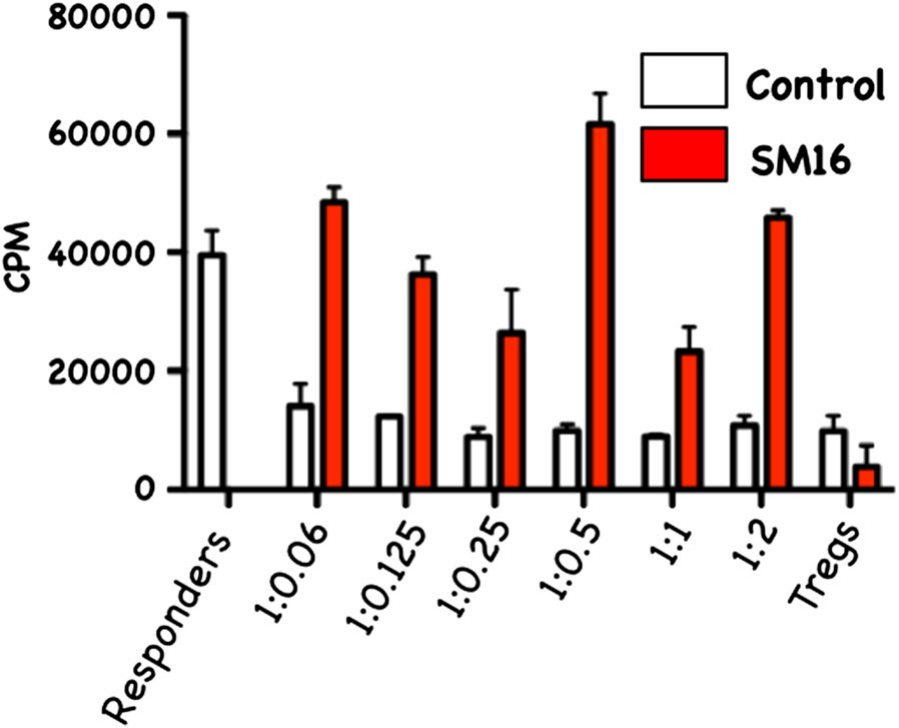
Treg having the ability to suppress the proliferation of naive T cells isolated from spleen of TBM BALB/c mice, fed control or SM16 diet for 19 days. CD4^+^CD25^+^ cells from donor mice were obtained by FACS sorting. The activity of isolated suppressor cells was tested by adding increasing ratios of CD4^+^CD25^+^ T cells to co-cultures of sorted CD4^+^CD25^−^ (responder cells) and APCs from naive mice in the presence of anti-CD3. After 48 h the plates were pulsed for 18 h with [^3^H]-thymidine. Subsequently, the cells were harvested on glass fiber filters and assessed for uptake of the labeled thymidine by liquid scintillation (3 independent experiments)

**Fig. 8:**
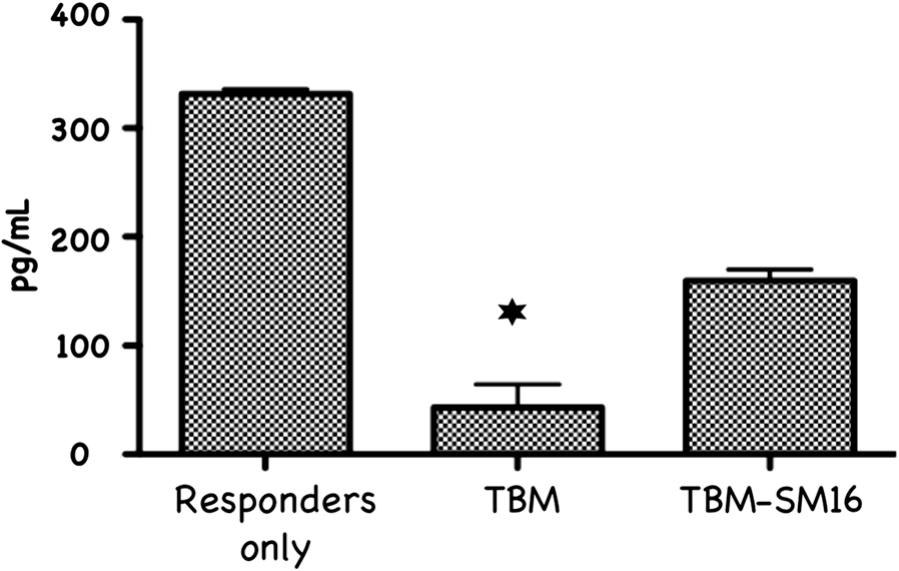
Effect of CD4^+^CD25^+^ T cells from TBM on IFN-γ production. CD4^+^CD25^+^ T cells from TBM either on control or SM16 diet were co-cultured with CD4^+^CD25^−^ responder T cells from naive donors (ratio: 1:1) and stimulated with irradiated APC and soluble anti-CD3 mAb. After 72 h supernatants were harvested from the co-cultures and were analyzed for IFN-γ concentrations. The amounts of IFN-γ production were evaluated in triplicate using OptEIA ELISA kit (Pharmingen). The presented value of IFN-γ production corresponds to 5 × 10^4^ cells. Bars represent mean values ± SEM (n = 3 from a single experiment). Significance between control and experimental groups were determined by Student’s t-test

SM16 treatment of tumor-bearing mice resulted in reduced suppressive activity of splenic CD4^+^CD25^+^ cells. As shown in Fig. 7, CD4^+^CD25^+^ T cells from spleen of TBM on control diet significantly inhibited proliferation of CD3-stimulated CD4^+^CD25^−^ responder cells as compared to SM16-fed mice. In addition to assessing the ability of CD4^+^CD25^+^ T cells to inhibit proliferation, we measured their effect on IFN-γ production by the CD4^+^CD25^−^ naive responders. Treg, responders and APC (irradiated CD90-depleted splenocytes) were plated at 1:1:1 ratio. As shown in Fig. 8, CD4^+^CD25^+^ T cells isolated from spleen of TBM on control diet, significantly reduced IFN-γ production by responder T cells. In contrast, co-incubation of responder T cells with CD4^+^CD25^+^ T cells isolated from spleen of SM16-fed TBM partially restored IFN-γ production.

## Discussion

CD4^+^CD25^+^FoxP3^+^ regulatory T cells are crucial to the maintenance of tolerance in normal individuals, and, so far gathered evidence indicates that presentation and progression of certain immunological disorders as well as the progression of cancer may be a function of Treg cell behavior [35]. Although, the factors regulating this cell population are still ill defined, it has been shown that TGF-β contributes directly to Treg differentiation and/or function [36]. Thus, TGF-β can be classified as a pro-tolerance agent, and, once present in the tumor microenvironment, it promotes immune tolerance to tumor cells by several mechanisms including expansion of the regulatory Treg compartment [12, 13]. This phenomenon has been recently implicated as one of the major factors contributing to immune evasion by tumors [37]. Thus, there has been a strong interest in finding strategies for blocking TGF-β signaling to overcome peripheral tolerance in order to enhance antitumor immunity. TGF-β signals through a heterodimeric receptor complex formed by association of TGF-βRI and TGF-βRII subunits with ligand. However, only binding of TGF- β to the TGF-βRI subunit of the receptor complex activates the intracellular kinase domain, which leads to the phosphorylation and activation of the Smad protein family and subsequent regulation of TGF-β-dependent gene expression [38]. Up-regulation of FoxP3 expression and acquisition of the regulatory phenotype by peripheral T cells is directly linked to TGF-β-induced Smad signaling [15]. However, TGF-β signaling blockade initiated by preventing the binding of ligand to the receptor via the use of antibodies, (antibodies neutralizing TGF-β peptide) or inhibitors of TGF-βRII activation has only been partially effective, and the major issue in reducing Treg effects is insufficient system saturation resulting in leakiness and incomplete inhibition of Smad signaling. For the above reason, the utilization of highly selective inhibitors of TGF-βR can guarantee a more robust inhibition of signaling and effectively prevent changes in gene expression, including the up-regulation of FoxP3. In the present work, we have identified SM16, a small molecule TGF-β signaling inhibitor, which binds the ATP pocket of TGF-βR1, and works as an anti-tolerance regulator of the Treg compartment. As shown herein, SM16 directly blocks the in vitro proliferative expansion of CD4^+^CD25^+^FoxP3^+^ Treg cells induced by TGF-β from BALB/c splenic CD4^+^CD25^−^ precursors (Fig. 1a, b), and from FoxP3eGFP splenic CD4^+^CD25^−^FoxP3^−^ precursors (Fig. 1c, d). Although SM16 inhibited the Treg inductive effect of TGF-β across multiple in vitro experiments (Fig. 1e), it did not block the expansion of Treg in vivo in transfer experiments of CD4^+^/GFP^−^ (FoxP3eGFP donors) donor cells transferred into lymphodepleted RagKO mice, or into lymphoreplete normal recipient mice. Previous reports have documented the in vivo effects of TGF-β to inhibit IL-2 production and T cell proliferation and differentiation [8, 39–41]. Thus, it was not unexpected that in vivo administration of SM16 would promote a broad homeostatic expansion of total cellularity in both the lymph nodes and spleens of recipient mice (Fig. 2); and, which resulted in statistically significant increases in absolute numbers of Treg (CD4^+^CD25^+^FoxP3^+^), activated T cells (CD4^+^CD25^+^FoxP3^−^) and naive (CD4^+^CD25^−^FoxP3^−^) T cells due to the blunting of autologous in vivo TGF-β effects. Attenuated TGF-β function may thus have resulted in concomitant increased systemic IL-2 production, and, simultaneously, abrogated Treg function, if not their frequency, in vivo (Table 1). Notably, in vivo SM16 administration stimulated the significant expansion of not only Tregs, but also a second “Treg-like” subpopulation (CD4^+^CD25^−^FoxP3^+^) in splenocytes and lymph node cells of lymphoreplete mice (Fig. 3a, b). Our studies therefore indicate that SM16 is not sufficient to inhibit the induction of the regulatory phenotype in activated cells and prevent expansion of Treg compartment in vivo. However, although SM16 did not reduce the in vivo frequency and absolute numbers of Treg cells, Treg from SM16 treated FoxP3eGFP animals showed significantly lower levels of suppression of CD3 stimulated T cell proliferation compared to Treg from naive untreated controls in a standard Treg functional assay (Fig. 4a). Similarly, the “Treg-like” (CD4^+^CD25^−^FoxP3^+^) subpopulation also showed significantly lower levels of suppression in a Treg functional assay (Fig. 4b). The anergy of Treg subpopulations in SM16 treated normal naive mice was also reflected in Treg subpopulations examined in SM16 treated TBM. Thus, although SM16 administration resulted in significantly inhibited tumor growth (Fig. 5) and concomitant increase in the CD4^+^CD25^−^FoxP3^+^ Treg-like subpopulation (Fig. 6a), it did not significantly increase the CD4^+^CD25^+^FoxP3^+^ Treg subpopulation in the spleen, lymph nodes or tumor microenvironment of TBM (Fig. 6b). Moreover, CD4^+^CD25^+^FoxP3^+^ Treg from TMB mice fed SM16 were significantly less suppressive in the standard Treg suppression assay than Treg from spleens of untreated TMB control mice (Fig. 7). Similarly, Treg from SM16 treated TBM were significantly less effective at suppressing IFN-γ secretion by CD3 activated T cells in vitro when compared to Treg from non-treated TBM control mice (Fig. 8). These findings may provide further explanation for the enhanced IFN-γ production and antitumor CTL activity in splenocytes from SM16-treated mice that we observed in our earlier studies [19, 26]. The blockade of TGF-β signaling has been shown to restore the frequency, persistence and cytotoxic activity among CD8^+^ T cells in various established tumor models, and may work in part by blocking Treg expansion and activation [42, 43]. Thus, our data suggest that while systemic blockade of TGF-βRI signaling may diminish the suppressive potential of Treg cells, it does so without substantially reducing their frequency or absolute numbers in SM16 treated mice. Unexpectedly, our study also revealed the expansion of a “Treg-like” CD4^+^ subpopulation which lacked CD25 expression but retained FoxP3 in splenocytes from SM16 treated normal control and TBM. Although these CD4^+^CD25^−^FoxP3^+^ cells showed diminished suppressor function comparable to Tregs (CD4^+^CD25^+^FoxP3^+^) in normal controls, we did not study them in SM16 treated TBM. However, Bonelli and co-workers have described significant increases in a CD4^+^CD25^−^FoxP3^+^ subpopulation which correlates with progression of disease in SLE patients [44, 45]. Their detailed phenotype analysis indicated this subpopulation more closely resembled regulatory T cells rather than activated T cells; and although they could suppress T cell proliferation in vitro they could not inhibit IFN-γ production [45]. The report by Bonelli was preceded by several earlier studies, which collectively indicated the importance of CD25 as a possible activation marker within Tregs [46–48]. Moreover, loss of CD25 expression is also thought to be primarily responsible for age-dependent functional decline of Treg cells [49, 50]. The underlying reasons for the observed change in distribution of CD25 expression need further investigation and are still unknown. The SM16 induced expansion of the CD4^+^CD25^−^FoxP3^+^ subpopulation, which also displayed a diminished capacity to suppress T cell proliferation, is of special interest. Previous reports have suggested TGF-β alone was sufficient in vitro and in vivo to induce first FoxP3 expression, and subsequently CD25^+^ expression on peripheral TCR activated Treg precursors [14, 15]. However, more recent studies suggest that IL-2 is also required for TGF-β to trigger naive CD4^+^CD25^−^ cells to become CD25^+^ and express FoxP3 and develop into fully functional Treg [38, 51]. Thus, it is tempting to speculate that the CD4^+^CD25^−^FoxP3^+^ subpopulation may represent an expanding precursor pool of Treg which have been arrested in their differentiation by the SM16 induced dysregulation of the previously described IL-2/TGF-β signaling interaction [52] which may play a role in inducing fully functional Tregs. This conclusion is further supported by the observation that majority of these cells reside within the proliferating Ki67^+^ compartment (unpublished data). This hypothesis is also strengthened by many reports that highly enriched polyclonal CD4^+^CD25^−^ T cells can convert to CD4^+^CD25^+^ T cells in vivo upon homeostatic proliferation in different lymphopenic models [53, 54]. These newly generated CD4^+^CD25^+^ T cells were shown to be phenotypically and functionally the same as naturally occurring Tregs.

## Conclusions

The present study demonstrated that systemic blockade of TGF-βR signaling using SM16 does not change in vivo Treg frequency, and results in the global expansion of absolute numbers of T cells, including Treg. Notably, the blockade of normal TGF-β activation is reflected primarily in reduced suppressive activity and the inability of Treg to inhibit IFN-γ production from effectors cells. Furthermore, tissue-selective expansion of a subpopulation of “Treg-like” cells (CD4^+^CD25^−^FoxP3^+^) suggests that SM16 induces a population of cells which might resemble dysfunctional Treg cells found in both mice and humans suffering from autoimmune disorder due to the loss of Treg functionality. Herein, we hypothesize that these cells may represent a pool of Treg precursors which are arrested in their normal differentiation pathway and are not fully functional. Better understanding of how these cells, which also occur in autoimmune individuals, may support antitumor immune response in tumor bearing mice and may lead to novel therapeutic approaches to combat immune evasion by tumors and other diseases.

## Data Availability

Not applicable

## Abbreviations

TGF-β: transforming growth factor-beta
Treg: regulatory T cell
TBM: tumor-bearing mice
IFN-γ: interferon-gamma

## References

[1] Massague J (2008). TGFbeta in cancer. Cell.

[2] Wrzesinski SH, Wan YY, Flavell RA (2007). Transforming growth factor-beta and the immune response: implications for anticancer therapy. Clin Cancer Res.

[3] Padua D, Massague J (2009). Roles of TGFbeta in metastasis. Cell Res.

[4] Tian M, Schiemann WP (2009). The TGF-beta paradox in human cancer: an update. Future Oncol.

[5] Bierie B, Moses HL (2010). Transforming growth factor beta (TGF-beta) and inflammation in cancer. Cytokine Growth Factor Rev.

[6] Rubtsov YP, Rudensky AY (2007). TGFbeta signalling in control of T-cell-mediated self-reactivity. Nat Rev Immunol.

[7] Shull MM, Ormsby I, Kier AB, Pawlowski S, Diebold RJ, Yin M, Allen R, Sidman C, Proetzel G, Calvin D (1992). Targeted disruption of the mouse transforming growth factor-beta 1 gene results in multifocal inflammatory disease. Nature.

[8] Gorelik L, Flavell RA (2002). Transforming growth factor-beta in T-cell biology. Nat Rev Immunol.

[9] Flavell RA, Sanjabi S, Wrzesinski SH, Licona-Limon P (2010). The polarization of immune cells in the tumour environment by TGFbeta. Nat Rev Immunol.

[10] Derynck R, Akhurst RJ, Balmain A (2001). TGF-beta signaling in tumor suppression and cancer progression. Nat Genet.

[11] Ikushima H, Miyazono K (2010). TGFbeta signalling: a complex web in cancer progression. Nat Rev Cancer.

[12] Bierie B, Moses HL (2006). Tumour microenvironment: TGFbeta: the molecular Jekyll and Hyde of cancer. Nat Rev Cancer.

[13] Walker MR, Kasprowicz DJ, Gersuk VH, Benard A, Van Landeghen M, Buckner JH, Ziegler SF (2003). Induction of FoxP3 and acquisition of T regulatory activity by stimulated human CD4^+^CD25^−^ T cells. J Clin Invest.

[14] Chen W, Jin W, Hardegen N, Lei KJ, Li L, Marinos N, McGrady G, Wahl SM (2003). Conversion of peripheral CD4^+^CD25^−^ naive T cells to CD4^+^CD25^+^ regulatory T cells by TGF-beta induction of transcription factor Foxp3. J Exp Med.

[15] Fantini MC, Becker C, Monteleone G, Pallone F, Galle PR, Neurath MF (2004). Cutting edge: TGF-beta induces a regulatory phenotype in CD4^+^CD25^−^ T cells through Foxp3 induction and down-regulation of Smad7. J Immunol.

[16] Huber S, Schramm C, Lehr HA, Mann A, Schmitt S, Becker C, Protschka M, Galle PR, Neurath MF, Blessing M (2004). Cutting edge: TGF-beta signaling is required for the in vivo expansion and immunosuppressive capacity of regulatory CD4^+^CD25^+^ T cells. J Immunol.

[17] Marie JC, Letterio JJ, Gavin M, Rudensky AY (2005). TGF-beta1 maintains suppressor function and Foxp3 expression in CD4^+^CD25^+^ regulatory T cells. J Exp Med.

[18] Li MO, Wan YY, Sanjabi S, Robertson AK, Flavell RA (2006). Transforming growth factor-beta regulation of immune responses. Annu Rev Immunol.

[19] Rausch MP, Hahn T, Ramanathapuram L, Bradley-Dunlop D, Mahadevan D, Mercado-Pimentel ME, Runyan RB, Besselsen DG, Zhang X, Cheung HK (2009). An orally active small molecule TGF-beta receptor I antagonist inhibits the growth of metastatic murine breast cancer. Anticancer Res.

[20] Li B, Lalani AS, Harding TC, Luan B, Koprivnikar K, Huan TuG, Prell R, VanRoey MJ, Simmons AD, Jooss K (2006). Vascular endothelial growth factor blockade reduces intratumoral regulatory T cells and enhances the efficacy of a GM-CSF-secreting cancer immunotherapy. Clin Cancer Res.

[21] Mahic M, Yaqub S, Johansson CC, Tasken K, Aandahl EM (2006). FOXP3^+^CD4^+^CD25^+^ adaptive regulatory T cells express cyclooxygenase-2 and suppress effector T cells by a prostaglandin E2-dependent mechanism. J Immunol.

[22] Polanczyk MJ, Carson BD, Subramanian S, Afentoulis M, Vandenbark AA, Ziegler SF, Offner H (2004). Cutting edge: estrogen drives expansion of the CD4^+^CD25^+^ regulatory T cell compartment. J Immunol.

[23] Yingling JM, Blanchard KL, Sawyer JS (2004). Development of TGF-beta signalling inhibitors for cancer therapy. Nat Rev Drug Discov.

[24] Hahn T, Akporiaye ET (2006). Targeting transforming growth factor beta to enhance cancer immunotherapy. Curr Oncol.

[25] Massague J (2000). How cells read TGF-beta signals. Nat Rev Mol Cell Biol.

[26] Garrison K, Hahn T, Lee WC, Ling LE, Weinberg AD, Akporiaye ET (2012). The small molecule TGF-beta signaling inhibitor SM16 synergizes with agonistic OX40 antibody to suppress established mammary tumors and reduce spontaneous metastasis. Cancer Immunol Immunother.

[27] Suzuki E, Kim S, Cheung HK, Corbley MJ, Zhang X, Sun L, Shan F, Singh J, Lee WC, Albelda SM (2007). A novel small-molecule inhibitor of transforming growth factor beta type I receptor kinase (SM16) inhibits murine mesothelioma tumor growth in vivo and prevents tumor recurrence after surgical resection. Cancer Res.

[28] Wallace A, Kapoor V, Sun J, Mrass P, Weninger W, Heitjan DF, June C, Kaiser LR, Ling LE, Albelda SM (2008). Transforming growth factor-beta receptor blockade augments the effectiveness of adoptive T-cell therapy of established solid cancers. Clin Cancer Res.

[29] Fu K, Corbley MJ, Sun L, Friedman JE, Shan F, Papadatos JL, Costa D, Lutterodt F, Sweigard H, Bowes S (2008). SM16, an orally active TGF-beta type I receptor inhibitor prevents myofibroblast induction and vascular fibrosis in the rat carotid injury model. Arterioscler Thromb Vasc Biol.

[30] Dexter DL, Kowalski HM, Blazar BA, Fligiel Z, Vogel R, Heppner GH (1978). Heterogeneity of tumor cells from a single mouse mammary tumor. Can Res.

[31] Kobie JJ, Wu RS, Kurt RA, Lou S, Adelman MK, Whitesell LJ, Ramanathapuram LV, Arteaga CL, Akporiaye ET (2003). Transforming growth factor beta inhibits the antigen-presenting functions and antitumor activity of dendritic cell vaccines. Can Res.

[32] Pulaski BA, Ostrand-Rosenberg S (1998). Reduction of established spontaneous mammary carcinoma metastases following immunotherapy with major histocompatibility complex class II and B7.1 cell-based tumor vaccines. Cancer Res.

[33] Wu RS, Kobie JJ, Besselsen DG, Fong TC, Mack VD, McEarchern JA, Akporiaye ET (2001). Comparative analysis of IFN-gamma B7.1 and antisense TGF-beta gene transfer on the tumorigenicity of a poorly immunogenic metastatic mammary carcinoma. Cancer Immunol Immunother.

[34] Gorelik L, Flavell RA (2000). Abrogation of TGFbeta signaling in T cells leads to spontaneous T cell differentiation and autoimmune disease. Immunity.

[35] Adeegbe DO, Nishikawa H (2013). Natural and induced T regulatory cells in cancer. Front Immunol.

[36] Liu VC, Wong LY, Jang T, Shah AH, Park I, Yang X, Zhang Q, Lonning S, Teicher BA, Lee C (2007). Tumor evasion of the immune system by converting CD4^+^CD25^−^ T cells into CD4^+^CD25^+^ T regulatory cells: role of tumor-derived TGF-beta. J Immunol.

[37] Whiteside TL (2012). What are regulatory T cells (Treg) regulating in cancer and why?. Semin Cancer Biol.

[38] Wan YY, Flavell RA (2007). ‘Yin-Yang’ functions of transforming growth factor-beta and T regulatory cells in immune regulation. Immunol Rev.

[39] Kehrl JH, Wakefield LM, Roberts AB, Jakowlew S, Alvarez-Mon M, Derynck R, Sporn MB, Fauci AS (1986). Production of transforming growth factor beta by human T lymphocytes and its potential role in the regulation of T cell growth. J Exp Med.

[40] Das L, Levine AD (2008). TGF-beta inhibits IL-2 production and promotes cell cycle arrest in TCR-activated effector/memory T cells in the presence of sustained TCR signal transduction. J Immunol.

[41] Das LM, Torres-Castillo MD, Gill T, Levine AD (2013). TGF-beta conditions intestinal T cells to express increased levels of miR-155, associated with down-regulation of IL-2 and itk mRNA. Mucosal Immunol.

[42] Chen ML, Pittet MJ, Gorelik L, Flavell RA, Weissleder R, von Boehmer H, Khazaie K (2005). Regulatory T cells suppress tumor-specific CD8 T cell cytotoxicity through TGF-beta signals in vivo. Proc Natl Acad Sci USA.

[43] Golgher D, Jones E, Powrie F, Elliott T, Gallimore A (2002). Depletion of CD25+ regulatory cells uncovers immune responses to shared murine tumor rejection antigens. Eur J Immunol.

[44] Bonelli M, Savitskaya A, Steiner CW, Rath E, Smolen JS, Scheinecker C (2009). Phenotypic and functional analysis of CD4^+^CD25^−^Foxp3^+^ T cells in patients with systemic lupus erythematosus. J Immunol.

[45] Bonelli M, von Dalwigk K, Savitskaya A, Smolen JS, Scheinecker C (2008). Foxp3 expression in CD4^+^ T cells of patients with systemic lupus erythematosus: a comparative phenotypic analysis. Ann Rheum Dis.

[46] Baecher-Allan C, Brown JA, Freeman GJ, Hafler DA (2001). CD4^+^ CD25high regulatory cells in human peripheral blood. J Immunol.

[47] Sakaguchi S, Sakaguchi N, Asano M, Itoh M, Toda M (1995). Immunologic self-tolerance maintained by activated T cells expressing IL-2 receptor alpha-chains (CD25). Breakdown of a single mechanism of self-tolerance causes various autoimmune diseases. J Immunol.

[48] Suri-Payer E, Amar AZ, Thornton AM, Shevach EM (1998). CD4^+^CD25^+^ T cells inhibit both the induction and effector function of autoreactive T cells and represent a unique lineage of immunoregulatory cells. J Immunol.

[49] Shimizu J, Moriizumi E (2003). CD4^+^CD25^−^ T cells in aged mice are hyporesponsive and exhibit suppressive activity. J Immunol.

[50] Nishioka T, Shimizu J, Iida R, Yamazaki S, Sakaguchi S (2006). CD4^+^CD25^+^Foxp3^+^ T cells and CD4^+^CD25^−^Foxp3^+^ T cells in aged mice. J Immunol.

[51] Zheng SG, Wang J, Wang P, Gray JD, Horwitz DA (2007). IL-2 is essential for TGF-beta to convert naive CD4^+^CD25^−^ cells to CD25^+^Foxp3^+^ regulatory T cells and for expansion of these cells. J Immunol.

[52] Tischner D, Wiegers GJ, Fiegl H, Drach M, Villunger A (2012). Mutual antagonism of TGF-beta and Interleukin-2 in cell survival and lineage commitment of induced regulatory T cells. Cell Death Differ.

[53] Curotto de Lafaille MA, Lino AC, Kutchukhidze N, Lafaille JJ (2004). CD25^−^ T cells generate CD25+ Foxp3^+^ regulatory T cells by peripheral expansion. J Immunol.

[54] Zelenay S, Lopes-Carvalho T, Caramalho I, Moraes-Fontes MF, Rebelo M, Demengeot J (2005). Foxp3^+^CD25^−^ CD4 T cells constitute a reservoir of committed regulatory cells that regain CD25 expression upon homeostatic expansion. Proc Natl Acad Sci USA.

